# Sulforaphane’s Multifaceted Potential: From Neuroprotection to Anticancer Action

**DOI:** 10.3390/molecules28196902

**Published:** 2023-10-01

**Authors:** Raymond A. Otoo, Antiño R. Allen

**Affiliations:** 1 Division of Radiation Health, University of Arkansas for Medical Sciences, 4301 West Markham, Suite 441B-2, Little Rock, AR 72205, USA; raotoo@uams.edu; 2 Department of Pharmaceutical Sciences, University of Arkansas for Medical Sciences, 4301 West Markham, Suite 441B-2, Little Rock, AR 72205, USA; 3 Neurobiology & Developmental Sciences, University of Arkansas for Medical Sciences, 4301 West Markham, Suite 441B-2, Little Rock, AR 72205, USA

**Keywords:** sulforaphane, antioxidant, cancer, chemotherapy

## Abstract

Sulforaphane (SFN) is a naturally occurring compound found in cruciferous vegetables such as broccoli and cauliflower. It has been widely studied for its potential as a neuroprotective and anticancer agent. This review aims to critically evaluate the current evidence supporting the neuroprotective and anticancer effects of SFN and the potential mechanisms through which it exerts these effects. SFN has been shown to exert neuroprotective effects through the activation of the Nrf2 pathway, the modulation of neuroinflammation, and epigenetic mechanisms. In cancer treatment, SFN has demonstrated the ability to selectively induce cell death in cancer cells, inhibit histone deacetylase, and sensitize cancer cells to chemotherapy. SFN has also shown chemoprotective properties through inhibiting phase I metabolizing enzymes, modulating phase II xenobiotic-metabolizing enzymes, and targeting cancer stem cells. In addition to its potential as a therapeutic agent for neurological disorders and cancer treatment, SFN has shown promise as a potential treatment for cerebral ischemic injury and intracranial hemorrhage. Finally, the ongoing and completed clinical trials on SFN suggest potential therapeutic benefits, but more research is needed to establish its effectiveness. Overall, SFN holds significant promise as a natural compound with diverse therapeutic applications.

## 1. Origin and Discovery

**Figure 1:**
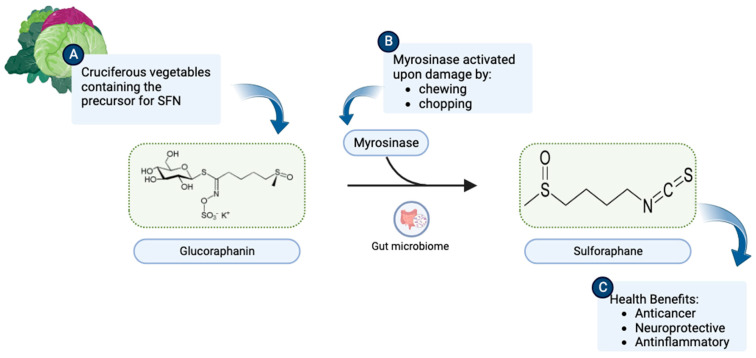
(**A**) Cruciferous vegetables are a rich source of glucoraphanin. (**B**) Upon chewing or chopping, the myrosinase enzyme present in plant tissues or intestinal flora catalyzes the breakdown of glucoraphanin to SFN (C_6_H_11_NOS_2_). (**C**) SFN consequently becomes available to exert health benefits. (Chemical structures of SFN and Glucoraphanin were sourced from their respective Wikipedia pages: https://en.wikipedia.org/wiki/Sulforaphane and https://en.wikipedia.org/wiki/Glucoraphanin.) This illustration was made with Biorender.com (accessed on 8 August 2023).

**Figure 2:**
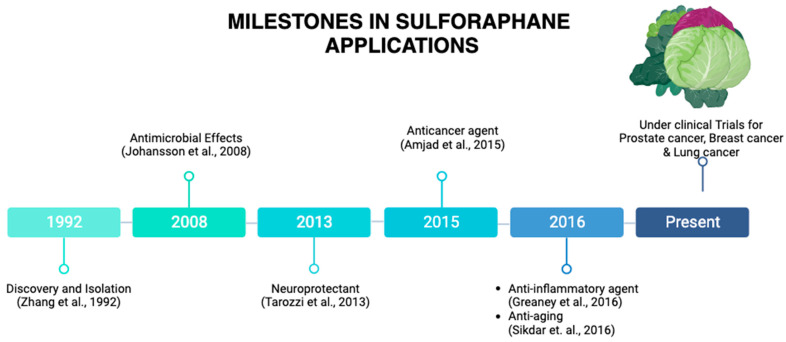
**Milestones in SFN applications.** SFN was discovered in 1992. A remarkable milestone has been reached, from its applications as an antimicrobial agent, a neuroprotective agent, an anticancer agent, and anti-inflammatory agent to currently being under clinical trials for prostate cancer, breast cancer, and lung cancer, among others. This illustration was made with Biorender.com (accessed on 8 August 2023) [2,8,9,10,11,12].

In the middle of the last century, sulforaphane (SFN; sulphoraphane in British English) was described as an antibiotic and was isolated from red cabbage and from hoary cress, a weed in rangelands of the western US [1]. It was first synthesized by Talalay and Zhang, who were the first to isolate it from broccoli [2]. SFN is a compound within the isothiocyanate (ITC) group of organosulfur compounds. ITCs are hydrolysis products of glucosinolates, secondary plant metabolites that are found in high concentrations in *Brassica* vegetables [3]. ITCs are known to be synthesized and stored as glucosinolates in plants and are released when damage to plant tissues occurs [4]. The most characterized ITC compound is SFN, the hydrolysis product of glucoraphanin, and it generally is found in high concentrations in broccoli (Figure 1) [5]. SFN occurs in broccoli sprouts, which have been shown to be 20–50 times more effective in chemoprevention than mature heads [6]. Among cruciferous vegetables, broccoli sprouts have the highest concentration the SFN precursor [7], hence broccoli sprouts are preferred over other crucifers as a chemoprotective agent. For this review, we will explore the multifaceted role of SFN including evidence that supports SFN’s neuroprotective effects, its potential as an anticancer agent, and its ability to act as a chemoprotective agent. Additionally, we will discuss the current state of clinical trials with SFN on some selected types of cancer and its potential to enhance the effectiveness of chemotherapy and radiation therapy. Figure 2 illustrates the journey of SFN from a promising naturally occurring compound to its status as a subject of ongoing research.

## 2. SFN as a Neuroprotective Agent

**Figure 3:**
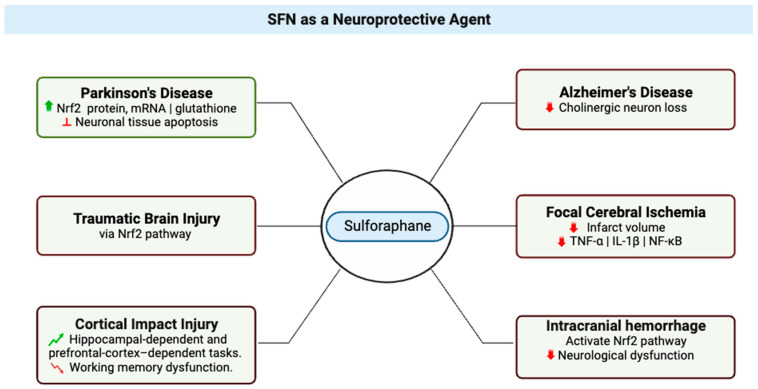
Multifaceted Neuroprotective Effects of Sulforaphane (SFN) in Diverse Neurological Conditions. The central node represents sulforaphane, while six distinct branches emanate from it, each depicting a specific condition where SFN exerts its therapeutic impact. Texts attached to each branch elaborate on the molecular mechanisms and outcomes associated with SFN’s effects in these conditions. Red signifies reduction or decrease in effects. Green indicates an increase or improvement in effects. This illustration was made with Biorender.com (accessed on 8 August 2023) [24,25,26,27,28,29,30,31].

Neuroprotection refers to the mechanisms and strategies used to defend the central nervous system (CNS) (Figure 3) against injury due to both acute (e.g., trauma or stroke) and chronic neurodegenerative disorders (e.g., dementia, Parkinson’s, Alzheimer’s, epilepsy) [13]; by extension, neuroprotective agents comprise a category of agents that generally are used to protect neuronal structure and/or function. Research on the neuroprotective effects of SFN began in 2004 with studies that showed its effects protecting neurons [14] and microglia [15] against oxidative stress via activation of nuclear factor erythroid 2-related factor 2 (Nrf2). The research literature is replete with studies that support the vital role played by the Nrf2 pathway in the neuroprotective effects of SFN [14,16,17,18,19,20,21], evidenced by lack of neuroprotection from toxins in Nrf2-knockout mice treated with SFN [22,23]. In a study of Parkinson’s disease that used a 6-hydroxydopamine-Parkinson’s disease mouse model, treatment of SH-SY5Y cells with SFN was found to have a protective effect on the neurons, which was attributed to the observed increases in active nuclear Nrf2 protein, Nrf2 mRNA, and total glutathione levels and inhibition of neuronal tissue apoptosis [24]. A group studying SFN effects in traumatic brain injury confirmed that SFN showed neuroprotection in spinal cord injury, and it may be an emerging therapeutic agent in this setting [25]. In a study that examined whether administration of SFN after cortical impact injury could improve the performance of rats in hippocampal-dependent and prefrontal-cortex-dependent tasks, SFN treatment was reported to improve performance in the Morris Water Maze task (i.e., decreased latencies during learning and platform localization during a probe trial) and to reduce dysfunction in working memory dysfunction (tested with the delayed match-to-place task) [26].

SFN also has been shown to exert neuroprotective effects in Alzheimer’s disease (AD) (Figure 3); in brains of mice with Alzheimer’s disease-like lesions, SFN ameliorated neurobehavioral deficits by reducing cholinergic neuron loss. SFN is a potent inducer of the Nrf2 antioxidant response element (ARE) pathway, which plays a major role in upregulating cellular defenses to oxidative stress [27]. In 2016, Zhou et al. reported that SFN exerted its neuroprotective effect in several ways, such as mTOR-dependent prevention of neuronal apoptosis, Nrf2-dependent reductions in oxidative stress, and restoration of normal autophagy [17]. Park et al. reported that with an in vitro model, SFN protected neuronal cells from Aβ42-mediated cytotoxicity and ameliorated proteasome activities [32]. A key factor in SFN’s neuroprotective effects is the compound’s effect on neuroinflammation [33,34]. Hernández-Rabaza et al. assessed whether treatment with SFN reduces neuroinflammation; they reported that treatment with SFN promoted differentiation of microglia from the pro-inflammatory M1 phenotype to the anti-inflammatory M2 phenotype and reduced activation of astrocytes in hyperammonemic rats to reduce neuroinflammation [35].

Epigenetics emerges as a pivotal mechanism underlying the neuroprotective potential of SFN. In a study involving mouse neuroblastoma N2a cells expressing human Swedish mutant Aβ precursor protein (N2a/APPswe cells), SFN was observed to induce Nrf2 expression by reducing DNA methylation levels at the Nrf2 promoter [20,36]. The activation of the Nrf2 ARE pathway, subsequently leading to the upregulation of key downstream elements such as NAD(P)H quinone oxidoreductase 1, heme oxygenase 1, and glutathione peroxidase 1, plays a pivotal role in countering oxidative stress [37]. Collectively, SFN’s ability to modify genetic expression, influencing a spectrum of detrimental or protective agents, translates into reduced cellular damage and the attenuation of harmful protein accumulation. This culminates in comprehensive neurological enhancements across various disease states and toxin exposures [38].

Additionally, a noteworthy aspect of SFN’s mechanism involves its capacity as a histone deacetylase (HDAC) inhibitor, warranting attention. Notably, the intricate interplay between biological mechanisms governing cancer transcends genetics and prominently involves epigenetics [39]. As an increasingly explored avenue, epigenetic modifications have garnered attention as a promising strategy for cancer prevention [40]. Central to this landscape are histone deacetylases (HDACs), pivotal orchestrators of epigenetic restructuring [41], which bear relevance in SFN’s anticancer effect [42]. SFN’s HDAC inhibition has been discerned across various cancer types including breast [42,43,44], colorectal [45], and prostate cancer [46,47]. Reinforcing this notion, Hossain et al. (2020) conducted a study elucidating the significance of HDAC inhibition in the context of SFN treatment for breast cancer. Through examination of MCF-7 cells, the investigation delineated the concerted influence of SFN, derived from cruciferous vegetables, and HDAC inhibitors like Trichostatin A (TSA) on gene expression patterns associated with 1,25(OH)2D3 activity. In this context, HDAC inhibition was identified as a critical enhancer of SFN’s impact, with distinct histone acetylation responses differentiating SFN and TSA treatments. The study thereby illuminates the intricate interplay between SFN’s diverse biochemical effects and its ability to modulate HDAC activity, ultimately elucidating the multifaceted molecular underpinnings of their synergistic anticancer effects [42]. In sum, the evolving understanding of SFN’s intricate interplay with epigenetic mechanisms, particularly HDAC inhibition, unveils its promising potential as an agent for combating cancer.

Furthermore, SFN’s potential as a neuroprotective agent extends to various neurological conditions, including focal cerebral ischemia (Figure 3), neuroinflammation, and intracranial hemorrhage. Focal cerebral ischemia arises from reduced blood flow to a specific brain region, resulting in changes in cerebral function [48,49]. Recent studies have probed the neuroprotective effects of SFN in the management of stroke [50,51]. Zhao et al. utilized a rodent common carotid artery/middle cerebral artery model and demonstrated that SFN reduced infarct volume after focal cerebral ischemia [28]. Additionally, SFN exhibited anti-inflammatory effects in this context, reducing pro-inflammatory cytokines and suppressing the expression of phospho-nuclear factor kappa-light-chain-enhancer of activated B cells (NF-κB) p65 [29]. These findings collectively underscore SFN’s potential as a therapeutic agent against cerebral ischemic injury.

Neuroinflammation, a pivotal factor in several neurological disorders, has also been a target of SFN’s neuroprotective effects [35,52,53,54,55]. Microglia, the resident immune cells of the brain [56], play a key role in neuroinflammation by secreting pro-inflammatory and anti-inflammatory mediators [57]. Studies in hyperammonemic rat models revealed that SFN promotes microglia differentiation from pro-inflammatory M1 to anti-inflammatory M2 phenotypes, mitigating neuroinflammation [35]. Furthermore, SFN’s anti-inflammatory effects in microglia were demonstrated by attenuated expression of neuroinflammatory proteins and reduced mitogen-activated protein kinase (MAPK) effector signaling [35]. SFN’s role in countering neuroinflammation was also evident in a study by Subedi et al., where it inhibited nitrite production and decreased the translocation of NF-κB and production of proinflammatory cytokines in microglial cells [53]. These cumulative findings highlight SFN’s potential as a therapeutic tool in managing neuroinflammatory diseases.

Intracranial hemorrhage (ICH), characterized by bleeding within the intracranial vault, presents a significant challenge in medical management [30,31,58,59]. Recent studies have illuminated the potential role of SFN in ICH treatment. SFN’s activation of the Nrf2-ARE signaling pathway was found to improve neurological dysfunction after ICH [30]. Additionally, SFN demonstrated efficacy in reducing oxidative stress and inflammation in ICH, opening avenues for further research [30,31].

Collectively, the extensive body of research underscores SFN’s multifaceted neuroprotective potential, ranging from its impact on oxidative stress, inflammation, epigenetic regulation, and beyond. As scientific understanding evolves, SFN continues to emerge as a promising candidate for the treatment and management of various neurological conditions, offering a beacon of hope in the realm of neuroprotection and therapeutic intervention.

## 3. SFN as an Anticancer Agent

In recent years, SFN has gained attention for its potential use as an anticancer agent. In a study in 2015, Ullah proposed three major factors that enhance the plausibility of clinical applications and the translational value. First, normal cells are relatively resistant to SFN-induced cell death [60], an important feature for potential anticancer agents, and recent in vitro work demonstrated that SFN suppressed metastasis of triple-negative breast cancer cells by targeting the RAF/MEK/ERK pathway [61]. Second, SFN has good bioavailability; it can reach high intracellular and plasma concentrations, and it has been detected in breast tissues after a single oral administration [62,63]. Finally, a study by Myzak et al. in 2007 provided evidence that histone deacetylase was inhibited after human subjects ingested 68 g of broccoli sprouts, indicating that SFN provides anticancer pharmacological effects at levels that humans can readily ingest.

Ullah proposed a mechanism by which SFN exerts its chemopreventive effects. In this model, low to moderate levels of reactive oxygen species (ROS) are active participants in cellular functions and act as signaling molecules that sustain cellular proliferation and differentiation and that activate responses to oxidative stress [64]. Under normal, unstressed conditions, the cellular NRF2 level is very low [65], but it significantly increases upon exposure to electrophilic chemicals or ROS [66]. In response to oxidative or electrophilic stress, Nrf2 stimulates antistress signaling and consequently inhibits carcinogenesis [67]. Under conditions of stress, Kelch-like ECH-Associated Protein 1 (KEAP1), which is a cytosolic inhibitor of Nrf2, is oxidized, leading to stabilization and translocation of Nrf2 into the nucleus, where the transcription factor activates expression of genes crucial to antioxidant defense [68].

Expanding the horizon of SFN’s impact reveals its significant potential across various cancer types. Studies by Livingstone et al. (2022) uncover SFN’s potential in prostate health, as elevated SFN levels were observed in men receiving glucoraphanin supplements [69]. Supporting this, Singh et al. (2019) demonstrated SFN’s ability to inhibit glycolysis in prostate cancer cells, suggesting therapeutic implications [70].

**Figure 4:**
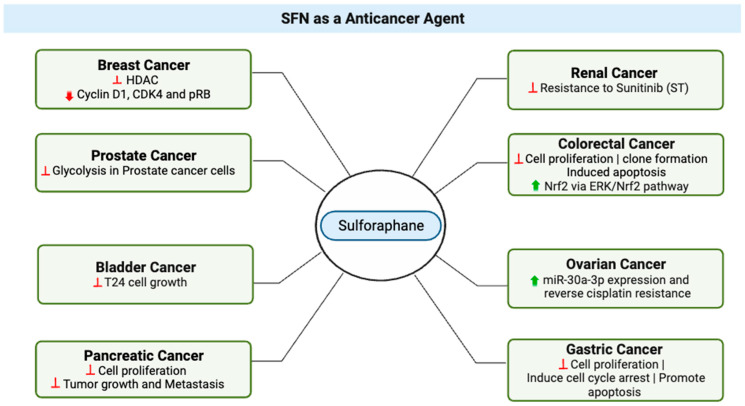
SFN’s Anticancer Effects Across Diverse Cancer Types. The various cancer types for which sulforaphane (SFN) exhibits potent anticancer properties. Red signifies reduction or decrease in effects. Green indicates an increase or improvement in effects. This illustration was made with Biorender.com (accessed on 8 August 2023) [44,70,71,72,73,74,75,76,77].

In bladder cancer (Figure 4), an in vitro study highlighted SFN’s dose-dependent effects on cell growth, presenting opportunities for targeted therapeutic strategies [71]. In this study, at higher concentrations, ranging from 10–160 μM and after 24 to 48 h of treatment, SFN demonstrated a significant inhibitory effect on T24 cell growth. However, it is important to consider that at lower doses, specifically 2.5 μM, SFN resulted in a slight increase in cell proliferation by 5.18–11.84% within a 6 to 48 h treatment window [71]. These results suggest that SFN’s effects on cell growth are dose-dependent, with potential implications for further research and development of targeted therapeutic strategies in the context of bladder cancer.

Interesting, insights from breast cancer cell research revealed that SFN orchestrates DNA methylation through the modulation of DNA methyltransferase and histone deacetylase levels, coupled with the downregulation of cyclin D1, CDK4, and pRB, thereby promoting breast cancer cell apoptosis [72].

Chen et al.’s comprehensive investigation (2018) showcased SFN’s efficacy in inhibiting pancreatic cancer cell proliferation, sensitizing cells to treatment, and affecting multiple cancer control hallmarks [73]. It inhibited clone formation and pancreatic cancer cell migration, induced apoptosis, and disrupted cell invasion in both low- and high-glucose environments, underscoring its multifaceted role. In the same study, in vivo experimentation using a transgenic mouse model further demonstrated SFN’s robust influence by significantly inhibiting tumor growth and metastasis.

Sunitinib (ST), an established therapy for renal cell carcinoma (RCC), faces limitations as a standalone treatment due to tumor reactivation and resistance [78]. To address this, an in vitro study investigated the combination of ST with sulforaphane (SFN). SFN emerged as a critical enhancer of ST’s efficacy by suppressing resistance in RCC cells, offering a potent approach to overcome ST monotherapy limitations. Short-term SFN application reduced cell numbers across diverse lines, sensitizing RCC cells to ST. Long-term SFN use exhibited greater effectiveness, particularly in 786O cells, where the ST-SFN combination outperformed SFN alone [74]. These findings underscore SFN’s significant anticancer potential in countering tumor reactivation and resistance, propelling further clinical research. Continued exploration of this dual therapy holds the promise of revolutionizing RCC management and advancing kidney cancer treatment.

A study aimed at investigating SFN’s capacity to counter resistance to cisplatin, a widely used ovarian carcinoma treatment, employed ovarian cancer cells. In this context, the investigation employed A2780 and IGROV1 cells, along with their cisplatin-resistant counterparts, A2780/CP70 and IGROV1-R10, to explore SFN’s potential in overcoming cisplatin resistance. The study unveiled SFN’s ability to effectively reverse cisplatin resistance by inducing DNA damage and enhancing intracellular cisplatin accumulation. Notably, SFN treatment resulted in a substantial elevation in miR-30a-3p expression, a microRNA that exhibited reduced levels in cisplatin-resistant cells [75]. The combined outcomes of this investigation strongly imply that SFN could heighten the efficacy of cisplatin against ovarian cancer cells. This enhancement is achieved through the upregulation of miR-30a-3p expression, triggering escalated DNA damage and heightened cisplatin accumulation within the cells.

For colorectal cancer, Hao et al.’s study (2020) demonstrated SFN’s potential as a chemopreventive agent (Figure 4), acting through the modulation of the ERK/Nrf2 pathway and impacting cell proliferation, apoptosis, and migration [76]. Additionally, SFN hindered the motility and migration of colorectal cancer cells. Mechanistically, SFN led to dose-dependent upregulation of nuclear factor, erythroid 2 like 2 (Nrf2) and UDP glucuronosyltransferase 1A (UGT1A) expression. This effect was mediated through the ERK/Nrf2 signaling pathway, as ERK inhibition attenuated SFN-induced upregulation of Nrf2 and UGT1A, along with mitigating increased intracellular ROS levels [13]. In sum, SFN emerges as a promising agent for colorectal cancer chemoprevention, acting through modulation of Nrf2-mediated detoxification and anti-proliferative pathways.

In the context of gastric cancer (GC), the intricate mechanisms of action of sulforaphane (SFN) have been explored to shed light on its anticancer properties (Figure 4). A recent study by Wang et al. (2021) has revealed that SFN can impede cell proliferation, induce cell cycle arrest, and promote apoptosis in GC cells [77]. Specifically, SFN treatment led to a pronounced reduction in cell viability, as evidenced by decreased colony-forming efficiency in BGC-823 and MGC-803 cell lines. Furthermore, SFN demonstrated its potential as an inducer of S phase cell cycle arrest, a critical regulator of cell proliferation, and showed promising apoptotic-inducing activity. Mechanistically, SFN achieved S phase arrest through the modulation of the p53-dependent p21-CDK2 axis, effectively inhibiting CDK2 expression while upregulating p53 and p21 levels. Additionally, SFN’s influence on the mitochondrial pathway emerged as a pivotal factor in its apoptotic effect, involving upregulation of Bax and cleaved-caspase-3 expression. Importantly, these findings, as documented by Wang et al. (2021) [77], provide valuable insights into SFN’s multifaceted role in suppressing GC cell growth and triggering apoptosis, thus highlighting its potential as a novel therapeutic agent for the treatment of gastric cancer.

These findings collectively highlight SFN’s diverse mechanisms of action, underscoring its potential as a versatile and potent anticancer agent across various malignancies. Its demonstrated impact on cancer cell proliferation, migration, and resistance presents promising opportunities for innovative therapeutic strategies and improved patient outcomes (Figure 4). Further research and clinical exploration are warranted to fully harness SFN’s potential in the realm of cancer therapy.

## 4. Chemoprotectant Properties of SFN

Cancer chemoprevention is defined as the use of dietary or pharmacological agents to prevent, block, or reverse the process of tumor development before clinical manifestation of the disease [79]. Chemoprotectants are natural or synthetic chemical compounds that can ameliorate, mimic, or inhibit the toxic or adverse effects of structurally different chemotherapeutic agents, radiation therapy, cytotoxic drugs, or naturally occurring toxins, without compromising the anticancer or antitumor potential of the chemotherapeutic drugs [80]. In vitro, SFN has been shown to be a potent chemopreventive agent and has been demonstrated to target multiple cellular mechanisms [81]. An in vivo study with an animal model (BALBc male mice) has shown that SFN prevented chemically induced cancers and inhibits tumor growth [82]. In a study of prostate cancer, SFN did not reduce the cytotoxic effects of drugs but, rather, strongly increased their anticancer efficacy against prostate cancer stem cells. In nude mice, combination treatment with SFN and a cytotoxic drug efficiently induced apoptosis and inhibited self-renewing potential, ALDH1 activity, clonogenicity, xenograft growth, and relapse of gemcitabine-treated tumor cells [83].

Inhibition of phase I metabolizing enzymes was reported by Langouet et al. as the primary mechanism of chemoprotection by SFN. A secondary mechanism has been proposed, in which phase II xenobiotic-metabolizing enzymes are modulated, and binding of carcinogens to DNA is directly inhibited [84]. As a result, cellular pro-inflammatory responses are suppressed, which inhibits formation of DNA adducts and reduces the mutation rate [84]. A tertiary chemoprevention mechanism also has been proposed, in which SFN abrogates tumorigenesis and progression of metastasis by targeting cancer stem cells in pancreatic and prostate cancer [83]. Royston et al. [72] reported that the combination of SFN with Withaferin A epigenetically reactivated tumor suppressor gene *p21* (also known as cyclin-dependent kinase inhibitor 1A).

## 5. Effects of SFN on Tumors, Chemotherapy, Radiation Therapy, and Cardiotoxicity

Laboratory and animal studies have provided evidence that SFN has anticancer properties, but more research is needed to understand its effects on humans. Studies have proposed that SFN may prevent the growth of certain types of tumors, such as breast and prostate tumors, by causing cell death and stopping the formation of new blood vessels, which are required for tumor growth. A study demonstrated that ROS activation of the p62-KEAP1-Nrf2 signaling pathway has a tumor-suppressive effect [85]. In stark contrast, Li et al. reported a study on bladder cancer cells that showed p62 promoted tumor growth by triggering the KEAP1-Nrf2 signaling pathway [86].

Research supports the proposition that SFN may improve the effectiveness of chemotherapy by increasing cancer cell sensitivity to the drugs used to treat them [87], which is known as “chemo-sensitization”. One way in which SFN may sensitize cancer cells to chemotherapy is by inhibiting the Nrf2 pathway [87]. Several studies have proposed that SFN can promote chemo-sensitization through the Nrf2 pathway [88,89,90]. Preclinical investigations have shown that SFN prevented mice from forming carcinogen-mediated mammary carcinogenesis, lung and gastric cancer, and colonic crypt foci [91]. The activation of Nrf2 leads to its binding to the ARE, this in turn results in the increased expression of antioxidant enzymes and detoxifying enzymes, consequently protecting normal cells from the toxic effects of chemotherapy while making cancer cells more susceptible to the drugs [92]. Another way that SFN may enhance the effectiveness of chemotherapy is by inducing cell death in cancer cells [93,94,95]. SFN can induce apoptosis (programmed cell death) in cancer cells and can inhibit the formation of new blood vessels that tumors need to grow, indirectly inducing cell death [96].

Some studies have suggested that SFN may have beneficial effects on the body after radiation therapy, although more research is needed to confirm these findings. Radiation therapy is a common treatment for cancer that uses high-energy radiation to kill cancer cells, but it can also damage healthy cells and tissues in the area being treated, leading to side effects such as skin irritation, fatigue, and a risk of developing secondary tumors. Preliminary studies have suggested that SFN may help protect healthy cells and tissues from the harmful effects of radiation [97]. For example, one study showed that SFN helped reduce inflammation and DNA damage in cells exposed to radiation, and results suggested that SFN may decrease the extent of radiation-induced skin damage in mice [98].

Cardiotoxicity is the occurrence of heart dysfunction as electric or muscle damage, resulting in heart toxicity [92]. Several studies have demonstrated the protective role of SFN in cardiotoxicity. For example, in a study by Bose et al. [99] with a breast cancer model in rats, they found that SFN reduced cardiac oxidative stress (a contributing factor to cardiotoxicity) induced by doxorubicin (DOX). When DOX was administered alone, only an 11% survival rate was observed as compared to a 62% survival rate observed when DOX was combined with SFN. This study also showed that combining SFN with DOX allowed for a 50% reduction in DOX dosage while maintaining its anticancer effects. In another study by Bai et al. [100], which monitored serum myocardial levels to assess cardiac injury markers in a treatment group receiving DOX, upon treatment with SFN, DOX-induced myocardial injury and inflammation was significantly reduced [99,100,101,102,103]. In sum, although studies have shown SFN’s protective role against cardiotoxicity induced by chemotherapy drugs such as DOX, further research is needed to fully understand the potential for SFN as a therapeutic agent for cancer treatment while mitigating its effects on heart function.

## 6. SFN on Metastasis

SFN exhibits anticancer properties at various stages of carcinogenesis in prostate, lung, colon, and breast cancers [104,105,106]. Early research has focused on the ability of SFN to activate nuclear factor (erythroid-derived 2)-like 2 (Nrf2). SFN was shown to be effective in preventing breast cancer at different stages of carcinogenesis by increasing the levels of antioxidants and phase II detoxifying enzymes via the activation of the nuclear factor erythroid 2-related factor 2 [107]. SFN has been shown to alter key mechanisms in vivo and in vitro which impact induction of cell cycle arrest and apoptosis and inhibition of histone deacetylase. SFN inhibits transforming growth factor-β1 (TGF-β1)-induced migration and invasion in human triple negative breast cancer (TNBC) cells [61]. Sulforaphane (SFE), an SFN derivative, has been shown to reduce TNBC proliferation by mediating ERG1/PTEN axis [107]. Sulforaphane exerted its anti-metastatic effects on non-small cell lung cancer through down-regulation of miR-616-5p, which was identified as a marker associated with risk of relapse and metastasis in patients [108]. SFN has been reported to inhibit histone deacetylase (HDAC) enzymes, alter histone acetylation, and affect gene regulation. Natural inhibitors of HDAC have received considerable interest as anticancer agents because of their ability to induce p21Cip1/Waf1, leading to cell cycle arrest and apoptosis [62]. SFN inhibits HDAC activity in prostate cancer cells, in mouse xenografts, and in human peripheral blood mononuclear cells [109]. In colon cancers, SFN blocks cells’ progression and angiogenesis by inhibiting HIF-1α and VEGF expression [110].

## 7. SFN Bioavailability and Pharmacokinetics

In mammals, SFN is metabolized rapidly via a conjugation reaction with glutathione. SFN is metabolized through the mercapturic acid pathway, starting with GSH conjugation by glutathione S-transferase and subsequently generating SFN-cysteine followed by SFN-N-acetylcysteine [111]. Pharmacokinetic studies in rodents have focused on either free SFN or its metabolite SFN-glutathione. Following consumption of broccoli, sulforaphane is excreted in urine predominantly as a conjugate with N-acetyl cysteine. In plasma, it has been found that approximately 50% of sulforaphane is found unconjugated with other thiols [104]. In rats, after an oral dose of 50 μmol of SFN, the plasma concentration of SFN can peak at 20 μM at 4 h and decline with a half-life of about 2.2 h [112]. SFN is well absorbed in the intestine, with an absolute bioavailability of approximately 82%. Elimination of was characterized by a long terminal phase; no major difference was evident in plasma concentrations between 6 and 24 h following intravenous administration or oral administration [113]. In mice fed with diets supplemented with 5 μmol/day and 10 μmol/day of SFN for 3 weeks, the steady-state levels of SFN in plasma and intestine reached 124–254 nM and 3–13 nmol/g of tissue [114]. In humans, given an oral capsule of 200 µmol, the peak plasma was reported to have a Cmax of 0.7 ± 0.2 µM at 3 h, with a half-life of 1.9 ± 0.4 h for elimination [115].

## 8. Ongoing and Completed Clinical Trials on SFN

**Figure 5:**
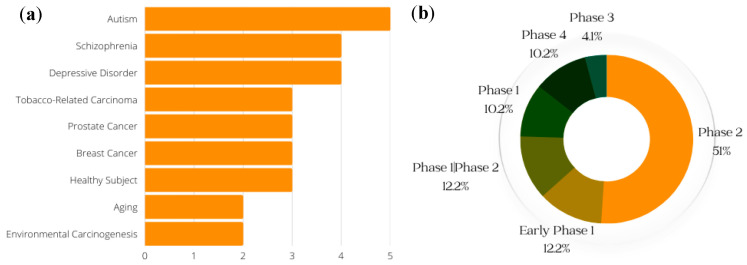
Conditions for which clinical trials with SFN have been registered. (Generated with data from ClinicalTrials.gov) (**a**) Conditions and respective numbers of clinical trials. The *x*-axis represents the number of studies that have recorded clinical trials with SFN and the conditions on the *y*-axis. (**b**) Breakdown of the phases of SFN clinical trials. This gives a general breakdown of how far SFN clinical trials have gone, with a majority of studies at the Phase 2 level.

Clinical trials are an important part of the research process, and drug development benefits from both favorable and unfavorable results. Even when studies do not yield the predicted outcomes, trial results can help point scientists in the correct direction [72]. (Clinical trials of SFN that were withdrawn are not included in the data presented here.) The conditions with the greatest number of ongoing or previous clinical trials on SFN, including breast cancer and prostate cancer, are shown in Figure 5a; the general phases of clinical trials on SFN (both past and current) are presented in Figure 5b. According to data obtained from ClinicalTrials.gov, a resource provided by the US National Library of Medicine, most clinical trials of SFN are in phase 2. A phase 1 trial (synonymous with “dose-escalation” or “human pharmacology” studies) is the first instance in which a new investigational agent is studied in humans. They are usually performed open-label and in a small number of “healthy” and/or “diseased” volunteers [83]. Phase 2 trials, also referred to as “therapeutic exploratory” trials, are usually larger than phase 1 studies and are conducted with a small number of volunteers who have the disease of interest [83].

Of the four trials involving schizophrenia, only one (NCT02810964) had results to report. The goal of this study was to determine whether symptoms of schizophrenia are reduced when standard antipsychotic medications are combined with SFN nutraceutical versus placebo. The primary outcome was a change (comparing beginning to end of treatment) in scores for the Positive and Negative Syndrome Scale [84]. Additionally, two phase 2 clinical trials (NCT00982319, NCT00843167) have examined effects of SFN in the setting of breast cancer. One of these trials (NCT00982319) revealed that SFN in broccoli-sprout extract resulted in an absolute change in the mean cellular proliferative rate, measured by Ki67 (a marker of active cell proliferation in the normal and tumor cell populations), from baseline to 14 days post-intervention [85]. The other trial (NCT00843167) investigated how treatment with broccoli-sprout extract affects women who have diagnoses of breast cancer, ductal carcinoma in situ, and/or atypical ductal hyperplasia. The results showed changes (comparing baseline to post-therapy) in ITC levels in urine samples, Ki67, and histone deacetylase activity in peripheral blood mononuclear cells [86].

## 9. Conclusions

In conclusion, this review stands as an invaluable resource, providing researchers with up-to-date insights into the latest advancements and developments in the multifaceted realm of Sulforaphane. By meticulously unraveling its historical journey, discovery, and expansive effects, we offer a comprehensive platform for researchers to grasp the current landscape of this remarkable natural compound abundant in *Brassica* vegetables, particularly broccoli sprouts. Across a diverse spectrum (Table 1), SFN showcases its potential for neuroprotection in neurological disorders, such as traumatic brain injury, Parkinson’s disease, Alzheimer’s disease, and epilepsy, while also unveiling its promising anticancer attributes, including potential chemoprotective and chemotherapeutic applications. Additionally, SFN’s exploration in managing conditions like intracranial hemorrhage and cerebral ischemic injury adds to its multifaceted profile. As clinical trials hint at the therapeutic prospects of SFN, ongoing research remains vital to solidify its efficacy. Thus, this mini review serves as a dynamic compass to guide researchers through the latest developments to expand the domain of Sulforaphane’s potential applications.

## Figures and Tables

**Table 1:** Summary of studies with SFN.

Topic	Article	Model	Effect
**Neuroprotectant**	Ladak et al., 2021 [14]	in vitro, cultured neuronal cells	Low doses of SFN in neuronal, astrocytes, and cocultures was neuroprotective
	Sandouka et al., 2021 [16]	in vitro, cortical cell cultures	SFN reduced neuronal cell death
		in vivo, temporal lobe epilepsy rat model	SFN exerted neuroprotective effects by increasing Nrf2 expression and related antioxidant genes, improved oxidative stress markers, and increased the total antioxidant capacity in both the plasma and hippocampus
	Zhao et al., 2019 [18]	in vitro, cultured HT22 mouse hippocampal cells	SFN protected HT22 cells against high glucose-induced injury
	Morroni et al., 2018 [24]	in vitro, cultured SH-SY5Y cells	SFN reduced neuronal apoptosis induced by 6-OHDA in SH-SY5Y cells
	Royston et al., 2018 [72]	in vitro, cultured breast cancer cell lines MCF-7 [ERα (+)] and the ERα (−) MDA-MB-231	SFN in combination with Withaferin A reactivated tumor suppressor gene p21
	Zhao et al., 2018 [20]	in vitro, cellular model of AD	SFN upregulated Nrf2 expression promoted the nuclear translocation of Nrf2 by decreasing DNA levels of the Nrf2 promoter, thus leading to antioxidative and anti-inflammatory properties
	Zhao et al., 2016 [21]	in vivo, animal model of SAH male Sprague–Dawley rats	Nrf2–ARE signaling pathway was activated in the basilar artery after SAH
	Benedict et al.,2012 [25]	in vivo, rat model of contusion SCI	SFN upregulated the phase 2 antioxidant response, decreased mRNA levels of inflammatory cytokines, and enhanced hindlimb locomotor function at the injury site
	Jazwa et al., 2011 [22]	in vivo, Nrf2-knockout mice and their wild type	SFN protected against MPTP-induced death of nigral dopaminergic neurons
	Mizuno et al., 2011 [19]	in vitro, primary neuronal cultures of rat striatum	SFN protected against H_2_O_2_- and paraquat-induced cytotoxicity
	Dash et al., 2009 [26]	in vivo, mouse model of TBI	SFN improved working memory, decreased oxidative damage in the brain
	Park et al., 2009 [32]	in vitro, cultured neurons with Aβ	SFN protected cells from Aβ_1–42_-mediated cell death in Neuro2A and N1E 115 cells
**Chemoprotectant**	Kallifatidis, G. et al., 2011 [82]	in vivo, BALBc male mice	SFN effectively inhibited tumor growth and increased the sensitivity of cancer cells
**Tumors**	Račkauskas et al., 2017 [87]	in vitro, culture CCC cells	Sulforaphane sensitized human cholangiocarcinoma to cisplatin
**Chemotherapy**	Choi et al., 2007 [93]	in vitro, cultured human prostate cancer cells	SFN induced cell death in human prostate cancer cells
	Wei et al., 2021 [97]	in vivo, RISI model (C57/BL6 mice)	SFN-mediated Nrf2 activation prevents radiation-induced skin injury
**Radiation Therapy**	Talalay et al., 2007 [96]	in vivo, human subjects and SKH-1 mice	SFN protected skin against damage by UV radiation
**Cardiotoxicity**	Bose et al., 2018 [99]	in vivo, cultured MCF 10A cells	SFN protected the heart from DOX toxicity
		in vitro, rat breast cancer model	SFN+DOX enhanced the activity in NRCM and MCF 10A cells
	Bai et al., 2017 [100]	in vivo, rat model (male Sprague–Dawley) of CHF	SFN reduced DOX-induced myocardial injury and inflammation
	Singh et al., 2015 [101]	in vivo, wild type 129/sv mice	SFN reduced DOX-induced cardiomyopathy mortality in mice
		in vitro, cultured rat H9c2 cardiomyoblast cells	SFN protected H9c2 cells from DOX cytotoxicity
	Li et al., 2015 [102]	in vitro, H9c2 rat myoblasts	SFN reduced ROS production and apoptosis induced by DOX in H9c2 cells
**Focal Cerebral Ischemia**	Li et al., 2022 [50]	in vivo, PSCI was modeled in wildtype (WT) and Nrf2 knockout (KO), male and female mice	Sulforaphane promoted white matter plasticity and improved long-term neurological outcomes after ischemic stroke
		in vitro, primary neuronal cultures	SFN reduced neuronal death
	Ma et al., 2015 [29]	in vivo, adult male Sprague–Dawley rats model of FCI	SFN inhibited cerebral ischemia-induced NF-κB pathway activation
	Subedi et al., 2020 [54]	in vitro, cultured BV2 microglial cells	SFN inhibited MGO-AGE-mediated neuroinflammation
**Neuro-Inflammation**	Wang et al., 2020 [55]	in vivo, rats	SFN improved LPS-induced neurocognitive dysfunction in rats
		in vitro, BV2 cells	SFN mitigated LPS-induced neuroinflammation through modulation of Cezanne/NF-κB signaling
	Subedi et al., 2019 [53]	in vitro, cultured BV2 cells	SFN exerted an anti-neuroinflammatory effect on microglia through JNK/AP-1/NF-κB pathway inhibition and Nrf2/HO-1 pathway activation
	Hernandez-Rabaza et al., 2016 [35]	in vivo, hyperammonemic rats	SFN reduced neuroinflammation
	Pan et al., 2023 [31]	in vivo, male BALB/c mice	SFN alleviated vascular remodeling
	Li et al., 2015 [102]	in vitro, H9c2 rat myoblasts	SFN reduced ROS production and apoptosis induced by DOX in H9c2 cells
**Intracerebral Hemorrhage**	Yin et al., 2015 [59]	in vivo, Sprague–Dawley rats of ICH	SFN decreased expression of Nrf2 and HO-1 in tissues surrounding hemorrhage and reduced perifocal inflammatory response
**Anticancer**	Zeng et al., 2011 [60]	in vitro, cultured colon cancer cells	SFN inhibited colon cancer cell (HCT116) proliferation
	Zhang et al., 2022 [61]	in vitro, cultured TNBR cells	SFN suppressed metastasis of triple-negative breast cancer cells
	Cornblatt et al., 2007 [63]	in vivo, female Sprague–Dawley rats	SFN distributed to the breast epithelial cells in vivo and exerts a pharmacodynamic action in these target cells

Abbreviations: DOX, doxorubicin; MGO-AGE, methylglyoxal-derived advanced glycation end-products; NF-κB, nuclear factor kappa-light-chain-enhancer of activated B cells; ROS, reactive oxygen species; SAH, subarachnoid hemorrhage; SFN, sulforaphane; SCI, traumatic spinal cord injury; TBI, traumatic brain injury; CCC, cholangiocarcinoma; RISI, radiation-induced skin injury; NRCM, Neonatal rat cardiac ventricular myocytes; CHF, chronic heart failure; PSCI, post-stroke cognitive impairment; FCI, focal cerebral ischemia; ICH, intracranial hemorrhage.

## Data Availability

Not applicable.
